# TSNAdb: A Database for Tumor-specific Neoantigens from Immunogenomics Data Analysis

**DOI:** 10.1016/j.gpb.2018.06.003

**Published:** 2018-09-15

**Authors:** Jingcheng Wu, Wenyi Zhao, Binbin Zhou, Zhixi Su, Xun Gu, Zhan Zhou, Shuqing Chen

**Affiliations:** 1 Institute of Drug Metabolism and Pharmaceutical Analysis and Zhejiang Provincial Key Laboratory of Anti-Cancer Drug Research, College of Pharmaceutical Sciences, Zhejiang University, Hangzhou 310058, China; 2 Hangzhou Institute of Innovative Medicine, College of Pharmaceutical Sciences, Zhejiang University, Hangzhou 310058, China; 3 College of Computer Science and Technology, Zhejiang University, Hangzhou 310013, China; 4 MOE Key Laboratory of Contemporary Anthropology, School of Life Sciences, Fudan University, Shanghai 200438, China; 5 Department of Genetics, Development and Cell Biology, Iowa State University, Ames, IA 50011, USA

**Keywords:** Neoantigen, Cancer immunotherapy, Somatic mutation, Human leukocyte antigen, Database

## Abstract

Tumor-specific **neoantigens** have attracted much attention since they can be used as biomarkers to predict therapeutic effects of immune checkpoint blockade therapy and as potential targets for **cancer immunotherapy**. In this study, we developed a comprehensive tumor-specific neoantigen **database** (TSNAdb v1.0), based on pan-cancer immunogenomic analyses of **somatic mutation** data and **human leukocyte antigen** (HLA) allele information for 16 tumor types with 7748 tumor samples from The Cancer Genome Atlas (TCGA) and The Cancer Immunome Atlas (TCIA). We predicted binding affinities between mutant/wild-type peptides and HLA class I molecules by NetMHCpan v2.8/v4.0, and presented detailed information of 3,707,562/1,146,961 potential neoantigens generated by somatic mutations of all tumor samples. Moreover, we employed recurrent mutations in combination with highly frequent HLA alleles to predict potential shared neoantigens across tumor patients, which would facilitate the discovery of putative targets for neoantigen-based cancer immunotherapy. TSNAdb is freely available at http://biopharm.zju.edu.cn/tsnadb.

## Introduction

Cancer somatic mutations and viral oncogenes can generate tumor-specific protein sequences that are entirely absent from normal human cells. These novel proteins may result in the formation of tumor-specific antigens (TSAs) [1]. As an important type of TSAs, neoantigens are generated by tumor-specific proteins, and presented by major histocompatibility complexes (MHCs) on cell surfaces through antigen presentation, where they can be recognized by T-cell receptors (TCRs) [2], [3]. Recently, neoantigens have attracted a large amount of attention, because they are potential biomarkers to distinguish tumor cells from normal cells. Neoantigens are of critical importance for cancer immunotherapy in the following two aspects. First, the neoantigen burden and quality can be used to predict therapeutic effects for immune checkpoint blockade therapy, such as blockage of programmed death-1 (PD-1) and cytotoxic T lymphocyte-associated antigen-4 (CTLA-4) [4], [5], [6]. Second, neoantigens can be used as potential targets for cancer immunotherapy, such as personalized cancer vaccines [7], [8] and adoptive cell therapy (ACT) [9]. Therefore, there is an urgent need to identify neoantigens accurately for cancer patients.

With the progress of cancer immunogenomics, several kinds of integrated software have been developed for tumor-specific neoantigen detection, such as TSNAD [10] and pVAC-seq [11]. The most critical function of such software is to predict the binding affinities between mutant peptides and human leukocyte antigen (HLA) alleles. To achieve this, a lot of well-acknowledged and popular tools, such as NetMHC [12], NetMHCpan [13], sNebula [14], and HLA-CNN [15], can be used. In addition, several databases can provide necessary information for the development of tools to predict the affinities between peptides and HLA alleles. For example, the Immune Epitope Database (IEDB) is an important immune-related database, providing a large amount of valuable and experimentally-validated information of immune epitopes [16]. The International Immunogenetics Information System (IMGT) offers information about antibodies, TCRs, MHCs, and so on [17]. Taking advantage of existing neoantigen prediction software, several neoantigen-related databases have been built. For example, TRON Cell Line Portal (TCLP) presents potential neoantigens of 1082 cancer cell lines [18]. The Cancer Immunome Atlas (TCIA) presents the relationship between tumor genotypes and immunophenotypes based on 20 solid cancers, and provides a quantitative index for immunotherapy response [19]. With the rapid growth of cancer genomics data, researchers are able to discover potential shared neoantigens across tumor patient populations [20], [21].

In this study, we developed a tumor-specific neoantigen database (TSNAdb v1.0) from pan-cancer immunogenomic analyses. Based on the 7748 tumor samples of 16 tumor types from The Cancer Genome Atlas (TCGA), we predicted the binding affinities between mutant/wild-type peptides and HLA class I molecules. Datasets we used include somatic mutation data of tumor samples from TCGA and the corresponding HLA allele data from TCIA. Two different versions of NetMHCpan, v2.8 [13], and v4.0 [22], were used for prediction. Furthermore, we also conducted extensive analyses and presented detailed information of potential neoantigens generated by somatic mutations, utilizing the related filtering tools embedded in TSNAD [10]. In addition, we employed the recurrent missense mutations in combination with the highly frequent HLA alleles to predict and analyze potential shared neoantigens. Our study would provide a platform to discover putative targets for neoantigen-based cancer immunotherapy.

## Database content and usage

### Data source

We collected somatic mutations and HLA alleles of 7748 tumor samples across 16 tumor types from TCGA (Release7.0, https://portal.gdc.cancer.gov) and TCIA (https://tcia.at/home), respectively. These tumor samples carry 972,187 missense mutations, among which 18,897 were found recurrently (at least three occurrences in all tumor samples). We selected the top 100 HLA alleles (frequency >0.5%) of 7748 tumor samples and combined them with the recurrent missense mutations to predict potential shared neoantigens. Moreover, we also extracted 13,459 recurrent missense mutations from 9155 samples derived from the International Cancer Genome Consortium (ICGC) (Release20, https://icgc.org/) and 16 highly frequent HLA alleles (frequencies >5%) from the 1000 Genome Project [23] for the prediction of potential shared neoantigens.

### Neoantigen prediction

We took the information on somatic mutations and HLA alleles of each tumor sample and employed NetMHCpan v2.8 [13] and NetMHCpan v4.0 [22] for neoantigen prediction, using the filtering tools embedded in our previously-developed software TSNAD [10]. All the peptides with 8–11 amino acids that contain missense mutations were extracted as mutant peptides, and the corresponding wild-type peptides were extracted as references. We collected the mutant peptides and HLA alleles with binding affinity IC_50_ < 500 nM (including strong binding with IC_50_ < 150 nM and weak binding with 150 nM < IC_50_ < 500 nM), without consideration of the binding level between their corresponding wild-type peptides and HLA alleles. We then clustered prediction results based on tumor types and calculated the frequencies of shared neoantigens. Compared with NetMHCpan v2.8, NetMHCpan v4.0 is trained based on both binding affinity data and mass spectrometry data, thus adopting stricter criteria for binding prediction. Consequently, 3,707,562 and 1,146,961 neoantigens were predicted by NetMHCpan v2.8 and v4.0, respectively, among which, 716,876 neoantigens were found in both predictions. The potential shared neoantigens based on recurrent mutations and highly frequent HLA alleles were predicted in the similar way.

### Web interface

**Figure 1:**
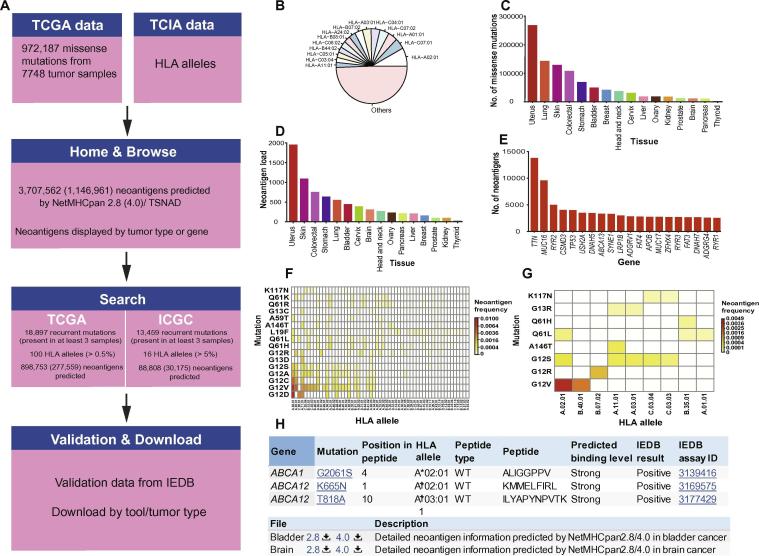
**An overview of the TSNAdb web interface** **A.** TSNAdb comprises five main components: (i) Home; (ii) Browse; (iii) Search; (iv) Validation, and (v) Download. The distribution of HLA alleles (**B)** and somatic missense mutations (**C)** extracted from TCGA and TCIA are listed. **D.** The average neoantigen loads across different tissues. **E.** Top 20 genes with predicted neoantigens in 7748 samples. **F.** The result of ‘Search’ page under the selection of “NetMHCpan2.8, *KRAS*, and TCGA”. **G.** The result of ‘Search’ page under the selection of “NetMHCpan2.8, *KRAS*, and ICGC”. **H.** Example of validation data from IEDB in ‘Validation’ page (top) and partial information on ‘Download’ page (bottom). The predicted binding level ‘Strong’ indicates strong binding with IC_50_ < 150 nM. TCGA, The Cancer Genome Atlas; TCIA, The Cancer Immunome Atlas; HLA, human leukocyte antigen; TSNAD, tumor-specific neoantigen detector; ICGC: International Cancer Genome Consortium; IEDB, Immune Epitope Database; WT, wild type.

To facilitate the utilization of TSNAdb, we have established a web interface to browse and analyze neoantigens. The web interface comprises five main pages (Figure 1A): (i) Home, (ii) Browse, (iii) Search, (iv) Validation, and (v) Download. In the following context, we exemplify the usage of TSNAdb with the results predicted by NetMHCpan v2.8.

In the ‘Home’ page, TSNAdb provides a statistical table about the database, including the number of projects, samples, HLA alleles (Figure 1B), mutations (Figure 1C), and predicted neoantigens of each tumor type. The table presents 3,707,562 potential neoantigens from the 7748 tumor samples across 16 tumor types. The average predicted neoantigen loads vary across different tumor types (Figure 1D). For instance, the average predicted neoantigen load for uterus cancer is 1957, which is 21 for thyroid cancer. Besides, users can get the detailed information about the predicted neoantigens of each tumor type by clicking on the tissue name in the table. The content of each tumor type includes top 20 HLA alleles and top 20 genes with predicted neoantigens in this tumor type, which are shown as two figures. All predicted neoantigens in this tumor type would be displayed below figures.

To further understand the distribution of neoantigens at gene level, users can retrieve neoantigens by feeding in a gene name in the ‘Browse’ page (Figure 1E). The retrieval results provide more functions than that indicated in each tumor type, such as sorting and searching within results, making it easier to search the most frequent neoantigens generated by each gene. For instance, the potential neoantigen based on *BRAF* V600E mutation and HLA-A03:01 exists in 117 tumor samples, and corresponding neoantigens based on several mutations of *KRAS* and *PIK3CA* are also shared in more than 40 tumor samples (Table 1).

**Table 1:** **Top 10 shared neoantigens of 7748 tumor samples from TCGA**

**Gene**	**Mutation**	**HLA allele**	**WT peptide**	**WT affinity (nM)**	**MT peptide**	**MT affinity (nM)**	**Frequency**
*BRAF*	V600E	A03:01	KIGDFGLATVK	94.09	KIGDFGLATEK	125.24	117/7748
*KRAS*	G12D	A02:01	KLVVVGAGGV	520.08	KLVVVGADGV	213.82	82/7748
*KRAS*	G12V	A02:01	KLVVVGAGGV	520.08	KLVVVGAVGV	111.87	71/7748
*KRAS*	G12V	A02:01	KLVVVGAG	17,690.28	KLVVVGAV	162.97	71/7748
*BRAF*	V600E	A11:01	KIGDFGLATVK	53.27	KIGDFGLATEK	45.20	68/7748
*PIK3CA*	H1047R	C07:01	AHHGGWTTKM	6742.50	ARHGGWTTKM	248.57	62/7748
*PIK3CA*	H1047R	C07:02	AHHGGWTTKM	2596.23	ARHGGWTTKM	217.76	56/7748
*PIK3CA*	E545K	A03:01	STRDPLSEITE	28,265.76	STRDPLSEITK	321.19	54/7748
*BRAF*	V600E	B57:01	FGLATVKSRW	128.34	FGLATEKSRW	246.23	41/7748
*BRAF*	V600E	B57:01	LATVKSRW	73.82	LATEKSRW	124.61	41/7748

*Note*: WT, wild type; MT, mutant. Amino acid residue changes caused by somatic mutations are indicated in red.

There would always be some newly discovered tumor patients with combinations of somatic mutations and HLA alleles absent from ‘Browse’ page. We thus provide neoantigen prediction results of all possible combinations (Cartesian product) of recurrent mutations (occurring at least three times in all samples) and highly frequent HLA alleles in ‘Search’ page. The data used for neoantigen prediction include 18,897 recurrent missense mutations and the top 100 HLA alleles (frequency >0.5%) of 7748 tumors from TCGA. Furthermore, we also employed the 13,459 recurrent missense mutations from ICGC and 16 HLA alleles with frequency >5% in the population collected in the 1000 Genome Project [23], for the prediction of potential shared neoantigens. Compared with the prediction results from real tumor samples, the frequencies of shared neoantigens predicted on recurrent mutations and highly frequent HLA alleles are similar (Table 2). The distribution of predicted shared neoantigens is displayed as shown in Figure 1F and G.

**Table 2:** **Frequency of the top 10 shared neoantigens predicted by recurrent mutations in combination with highly frequent HLA alleles from TCGA**

**Gene**	**Mutation**	**HLA allele**	**Expected frequency**	**Observed frequency**
*BRAF*	V600E	A03:01	1.55%	1.51%
*KRAS*	G12D	A02:01	1.01%	1.06%
*PIK3CA*	H1047R	C07:01	0.73%	0.80%
*PIK3CA*	E545K	A03:01	0.68%	0.70%
*PIK3CA*	E542K	A03:01	0.44%	0.44%
*TP53*	R248W	A02:01	0.33%	0.34%
*TP53*	R273C	A02:01	0.29%	0.31%
*TP53*	R248Q	C07:02	0.25%	0.23%
*TP53*	Y220C	A02:01	0.24%	0.19%
*PIK3CA*	R88Q	C07:02	0.16%	0.17%

*Note*: Expected frequency indicates the frequency of shared neoantigens predicted by recurrent mutations in combination with highly frequent HLA alleles. Observed frequency, the frequency of shared neoantigens in 7748 tumor samples.

Besides, we present experimentally-validated data for the predicted neoantigens in the ‘Validation’ page (Figure 1H), according to the binding level between peptides and HLA alleles. Limited by the availability of binding data between mutant peptides and HLA alleles, all the validation data derived from IEDB is for wild-type peptides and HLA alleles [16].

TSNAdb v1.0 (http://biopharm.zju.edu.cn/tsnadb/) is freely available for all academic users. Users can download data from the ‘Download’ page (Figure 1H), according to tumor types and the prediction tools chosen.

### Case study

The major function of our database is to provide potential neoantigens of various tumor types and shared neoantigens across tumor patient populations. Therefore, we further provide statistical analyses of neoantigen prediction results in each tumor type. Here, we take the results of bladder cancer predicted by NetMHCpan v2.8 as an example to demonstrate the utilization of TSNAdb.

**Figure 2:**
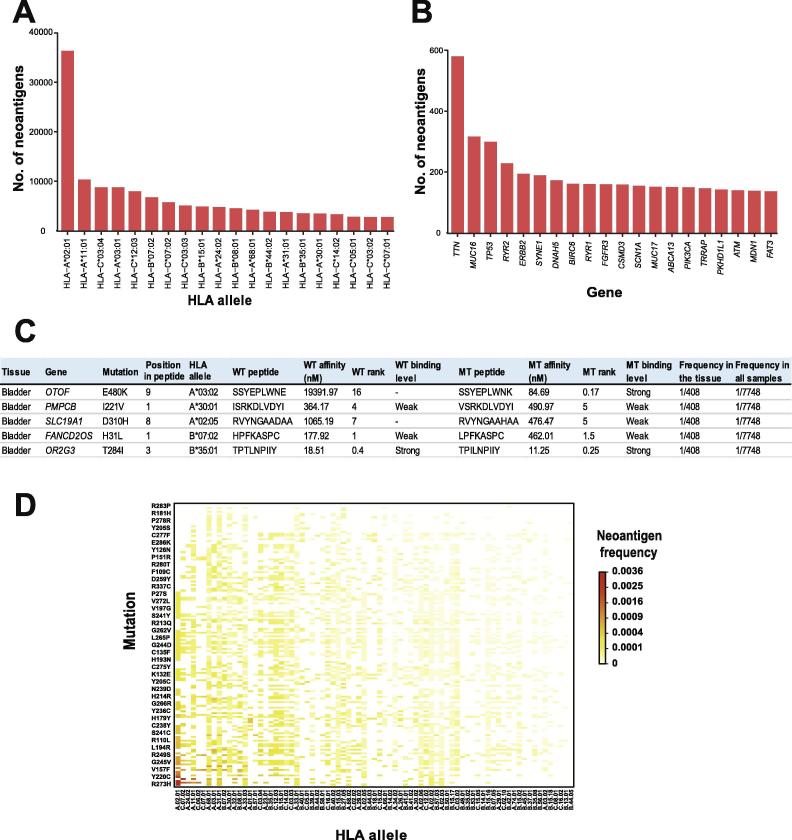
**Example applications of predicted neoantigens for bladder cancer and the gene *TP53*** Top 20 HLA alleles (**A**) and genes (**B**) with predicted neoantigens are displayed in the page using bladder cancer as an example, with the detailed neoantigen information listed (**C**). The binding level ‘Strong’ indicates strong binding with IC_50_ < 150 nM, ‘Weak’ indicates weak binding with 150 nM < IC_50_ < 500 nM, ‘-’ indicates non-binding with IC_50_ > 500 nM. **D.** Distribution of the predicted neoantigens for all combinations of recurrent mutations of *TP53* and the highly frequent HLA alleles according to the TCGA dataset. The color gradient indicates the frequencies of potential shared neoantigens for the specific combinations of somatic mutations and HLA alleles. All the data shown are predicted by NetMHCpan v2.8. HLA, human leukocyte antigen; TCGA, The Cancer Genome Atlas; WT, wild type; MT, mutant.

There are 408 tumor samples for bladder cancer, with 106 different HLA alleles and 49,537 missense mutations. From these tumor samples, we obtain 182,756 predicted neoantigens. We present the top 20 HLA alleles, top 20 genes, and detailed neoantigen information in the web page (Figure 2A–C). According to the number of predicted neoantigens presented by each HLA allele, the top three HLA alleles are A02:01, A11:01, and C03:04, which account for 19.5%, 5.6%, and 4.7% of the total HLA alleles, respectively. These three HLA alleles also show >5% frequency in the 1000 Genome Project [23]. According to the number of predicted neoantigens generated by each gene, top three genes are *TTN*, *MUC16*, and *TP53*, which have 584, 318, and 270 neoantigens, respectively. In these genes, *TTN* and *MUC16* encode large proteins with numerous random mutations, whereas *TP53* is the most famous tumor suppressor gene with lots of recurrent mutations. The most recurrent mutation of *TP53* is R248Q, which exists in 17 out of 408 bladder cancer patients. The mutant peptide arising as a consequence of *TP53* R248Q mutation could bind to HLA-C07:02 and be presented as a potential neoantigen in four patients, which is the most frequent neoantigen in bladder cancer. If a bladder cancer patient carries the same mutation and HLA allele with existing patients, such as *TP53* R248W and HLA-A02:01, the corresponding neoantigen can be retrieved from the ‘Browse’ page directly. And the potential neoantigens can be used for experimental validation, which would facilitate the following cancer immunotherapy. If the combination of HLA allele and *TP53* mutation of this patient is absent in the existing samples, users can try to retrieve it in the ‘Search’ page. For instance, the combination of *TP53* R273H and HLA-A02:01 is absent in existing bladder cancer patients but can be retrieved in the ‘Search’ page, which provides the predicted neoantigens generated by all combinations of recurrent *TP53* mutations and highly frequent HLA alleles (Figure 2D). There are 155 types of recurrent *TP53* mutations that can generate at least one potential neoantigen presented by highly frequent HLA alleles, and 130 of these mutations could be presented by at least ten highly frequent HLA alleles. For instance, peptides generated by *TP53* G105C mutation are predicted to bind to 47 different HLA alleles. The most frequent potential neoantigen is generated by *TP53* R273H (0.86%) and HLA-A02:01 (41.4%), which shows the frequency of 0.36%.

## Perspectives and concluding remarks

In this study, we developed a comprehensive database named TSNAdb for tumor-specific neoantigens based on 7748 tumor samples of 16 tumor types from TCGA. This database provides detailed affinity information between mutant/wild-type peptides and HLA alleles, and the frequencies of neoantigens shared by tumor samples of each tumor type and pan-cancer. Furthermore, this database also provides potential shared neoantigens generated from all possible combinations of recurrent mutations and highly frequent HLA alleles. The information provided by the database could facilitate the subsequent experimental design and validation and the discovery of potential targets for cancer immunotherapy. Compared with other existing neoantigen-related databases, such as TCIA, TSNADB provides the HLA binding information of both mutant peptides and wild-type peptides, which could be used for evaluating the differential agretopicity index (DAI), the difference of HLA binding affinity between mutant and wild-type peptides [24]. Besides, users could search neoantigens at the gene level and obtain the potential shared neoantigens generated from all possible combinations of recurrent mutations and highly frequent HLA alleles from TSNAdb, which makes the database more user-friendly and comprehensive. In the future, we would expand our work from the following three aspects. In terms of the data, we would collect more samples from not only TCGA, but also other cancer databases such as ICGC or published literatures, for more comprehensive combination of HLA alleles and somatic mutations. In terms of the methods, we would apply more state-of-the-art methods on the neoantigen prediction and update our prediction software to improve the accuracy. In terms of the evaluation metrics, we would employ more well-acknowledged metrics to evaluate predicted neoantigens, *e.g.*, neoantigen quality indicating the probability for TCR recognition [25], as well as DAI [24].

## Authors’ contributions

ZZ and SC conceived of the idea and supervised the study. ZS and XG participated in the design of the study. JW constructed and maintained the database and web interface, performed the data analysis. ZZ wrote the program and WZ designed the system architecture. WZ participated in the data analysis; ZS participated in the data acquisition; BZ participated in the statistical analysis. JW, BZ, and ZZ wrote the manuscript. All authors have read and approved the final manuscript.

## Competing interests

The authors have declared no competing interests.
